# Cooperation of Cancer Stem Cell Properties and Epithelial-Mesenchymal Transition in the Establishment of Breast Cancer Metastasis

**DOI:** 10.1155/2011/591427

**Published:** 2010-12-23

**Authors:** Tetsu Hayashida, Hiromitsu Jinno, Yuko Kitagawa, Masaki Kitajima

**Affiliations:** ^1^Department of Surgery, Keio University School of Medicine, 35 Shinanomachi, Shinjyuku, Tokyo 160-8582, Japan; ^2^Department of Surgery, International University of Health and Welfare, 1-4-3 Mita, Minatoku, Tokyo 108-8329, Japan

## Abstract

Epithelial-mesenchymal transition (EMT) is a multistep process in which cells acquire molecular alterations such as loss of cell-cell junctions and restructuring of the cytoskeleton. There is an increasing understanding that this process may promote breast cancer progression through promotion of invasive and metastatic tumor growth. Recent observations imply that there may be a cross-talk between EMT and cancer stem cell properties, leading to enhanced tumorigenicity and the capacity to generate heterogeneous tumor cell populations. Here, we review the experimental and clinical evidence for the involvement of EMT in cancer stem cell theory, focusing on the common characteristics of this phenomenon.

## 1. Introduction

Relapse and resultant metastatic spread to distant sites of malignant neoplasms remain the leading cause of mortality associated with cancer [1, 2]. The classical metastatic cascade includes intravasation by cancer cells, their circulation in the lymph and blood vascular systems, extravasation, and growth into metastatic foci [3, 4]. However, metastasis is considered to be an inefficient process, since only very few cells among the numerous cancer cells in the circulation have the ability to invade and form distant nodules [5]. In the past decade, two different concepts related to solid tumor progression have emerged and been intensively studied to explain these complicated phenomena. There is a growing understanding that epithelial-mesenchymal transition (EMT) can contribute to invasive and metastatic tumor growth. This process is considered to ultimately promote cancer cell progression through the basement membrane and invasion into the surrounding microenvironment, such as the lymph and blood vascular systems, contributing to intra- or extravasation [6–8]. On the other hand, there is increasing data to support the hypothesis that most tumors include a minor subpopulation of cells with distinct properties similar to somatic stem cells, which are referred to as cancer stem cells (CSCs) or tumor-initiating cells. CSCs have been reported to have enhanced tumorigenicity, compared with the majority of tumor cells from the same tumor, and the capacity to generate heterogeneous tumor cell populations [9–11]. Initially, these concepts were individually studied; however, Mani et al. suggested that there may be an association between EMT and the gain of CSC properties in breast cancer cells in 2008 [12], and another group reported that gene expression patterns of CSC-associated pathways were involved in EMT [13]. Here, we review recent studies of CSCs and the relationship with EMT and we consider what implications this correlation may have for our ability to explain aspects of tumor progression including distant metastasis.

## 2. Role of EMT in Tumor Metastasis

EMT was originally defined as a latent embryonic process causing epithelial cells to lose their epithelial behavior and acquire the properties of mesenchymal cells [14, 15]. This is a multistep process in which cells obtain molecular alterations that cause dysfunctional cell-cell adhesive interactions, loss of cell-cell junctions, and restructuring of the cytoskeleton; all of which result in the loss of apical polarity and the acquisition of a more spindle-shaped morphology [16–21]. This process is an important part of early development. For example, in vertebrates, this process facilitates the formation of a three-layered embryo by gastrulation [22, 23] and the mesenchymal phenotype allows migration to the proper site for organ formation [24]. In neoplasia, a comparable process is supposed to arise on the tumor front, allowing for cellular invasion and eventual metastatic dissemination of cancer cells [17, 25, 26]. 

EMT in tumor progression can be induced by several cytokines and chemokines, including transforming growth factor-*β* (TGF*β*) [27]. An increasing constitutive production and release of TGF*β* by tumor cells leads to the activation of the TGF*β* signaling pathway in an autocrine fashion, which results in an EMT state with a highly invasive and metastatic phenotype [28–30]. As significant evidence of EMT *in vivo*, recently, Giampieri and colleagues used elegant intravital imaging studies to visualize either collective or single-cell migration of cancer cells. They showed that TGF*β* signaling could drive a switch to single-cell migration without cell-cell attachment and that the mode of migration determined the way the tumor spread [31].

Exogenous expression of many developmentally important transcription factors is also known to induce EMT. Transcriptional repression of E-cadherin is mediated by members of the Snail/Slug family and Twist, and their critical role in EMT was confirmed by studies of ablation of Snail* in vivo* [32]. Both Twist and Snail promote tumor cell metastasis with no apparent stimulation of primary tumor growth. Snail even attenuates the cell cycle, rather than promoting proliferation, suggesting that these EMT regulators are also convincing metastasis genes [33–35]. 

Accordingly, activation of these processes in cancer cells significantly increases their metastatic potential, but some argument still exists as to whether EMT is a sufficient condition for cancer metastasis. Evidence for the role of EMT is complicated by the fact that at the secondary site the metastatic cells likely change those cellular phenotype to show heterogeneity, permitting colonization of the distant site. Several lines of experimental result have accumulated that indicate mesenchymal-epithelial transition (MET) may be important for the latter stages of metastasis, when cancer cells regenerate complex growth that recapitulate the histopathological complexity of the primary tumors from which they arose [36, 37]. Recent publications have demonstrated that MET is essential for the reprogramming of fibroblasts to induced pluripotent stem cells [38], suggesting the correlation between the reprogramming process in some cases and repression of the EMT program. Another study has shown that CSCs are necessary for homeostasis of metastatic cells that would require the reversion of EMT [39]. Therefore, in the course of cancer progression, EMT may be a transient and reversible process and not only EMT but also pluripotent roles in epithelial plasticity may be essential for the establishment of cancer metastasis.

## 3. Cancer Stem Cell Theory of Tumor Metastasis

The hypothesis that the majority of solid tumors contain a small subpopulation of cells with distinct properties similar to somatic stem cells is supported by both basic and clinical studies. This is a not-so-recent hypothesis that began with the discovery of a cell capable of initiating human acute myeloid leukemia. The population of cells that possessed the potential for self-renewal and differentiation was assumed to be a leukemia stem cell [40]. In 2003, Al-Hajj and colleagues for the first time isolated a CD44^+^/CD24^−/low^) subpopulation of breast cancer cells and showed that the cells in this population produced tumors in a xenograft model more effectively than did the majority population of tumor cells [9]. CD44^+^/CD24^−/low^cells were subsequently designated as CSCs, and this discovery has enhanced investigation of CSCs as the metastatic component of cancer, especially in breast cancer. Conceptually, CSCs must be engaged in the metastatic process if they are the only subset of cells capable of initiating new tumor growth. If that is the case, metastatic cells in the circulation should have the tumorigenic capacity necessary to induce tumor initiation at a distant metastatic site. This hypothesis was partly supported by reports showing that CD44^+^/CD24^−/low^ status of primary tumor was significantly correlated with distant-metastatic-free survival [41, 42], although the clinical relevance of the CD44^+^/CD24^−/low^ phenotype in primary breast cancer is still controversial. Furthermore, CD44^+^/CD24^−/low^ breast cancer cells expressed high levels of genes related to metastasis and induced lung metastasis *in vivo* [43]. In addition, analysis of genetic profiles confirmed that CD44^+^ breast cancer cells are enriched with stem-cell markers and display activated TGF*β* signaling and poor clinical outcomes [44]. 

The expression of aldehyde dehydrogenase 1 (ALDH1) has also been shown to be a CSC marker, and CSCs have been isolated from primary breast cancer using an ALDEFLUOR assay [45]. A recent study demonstrated that ALDH1 expression can be an independent prognostic factor for predicting metastasis in inflammatory breast cancer and that CSCs have the ability to reconstitute the heterogeneity of the primary tumor at the metastatic site [46]. ALDH1 was also identified as a predictor of poor prognosis in lung and bladder cancer [47, 48].

In the small and large intestine, leucine-rich repeat-containing G protein-coupled receptor 5 (LGR5) was identified as a marker for stem cells [49] and deletion of APC in LGR5-expressing cells induced their transformation within days, suggesting that LGR5 might be a limited population of CSCs [50]. From a clinical point of view, we recently showed that LGR5 was markedly overexpressed in the majority of advanced colorectal cancers (CRCs) compared with normal mucosal tissue [51]. This LGR5 expression, which was variable among CRC cases, correlated significantly with lymph node metastasis, suggesting the involvement of LGR5 in the metastatic process.

## 4. Common Characteristics in EMT and Tumor-Initiating Ability

### 4.1. TGF*β* Signaling Pathway

Several studies have indicated that EMT inducers can make cancer cells become more tumorigenic [7], giving rise to the hypothesis that tumor cells can transiently acquire stem cell-like properties as a consequence of EMT. Mani and colleagues performed *in vitro*-based experiments and reported that the induction of EMT in human mammary epithelial cells resulted in the acquisition of mesenchymal morphology and the expression of mesenchymal markers. This phenotypic EMT change increased the CD44^+^/CD24^−/low^ subpopulation, which exhibited the properties of stem cells, such as enhanced mammosphere-forming ability and differentiation into myoepithelial or luminal epithelial cells. They also demonstrated that transformed human mammary epithelial cells showed effective tumor-initiating ability with induction of EMT [12]. In that report, induction of EMT that resulted in the enrichment of CSCs was conducted by activation of the TGF*β* pathway, as well as by ectopic expression of the transcription factors Snail, Slug, and Twist.

Generally, activation of the TGF*β* pathway inhibits tumorigenesis; however, it is also known that the TGF*β* pathway cooperates with other pathways to assist tumorigenesis in the malignant state [52, 53]. Genetic signatures that predict poorer prognosis for primary breast cancer patients have been examined by comparing the gene expression profiles of CD44^+^/CD24^−/low^ cell populations with other populations [42, 44]. Following up on the above observations on the TGF*β* pathway, one of these studies, which performed SAGE profiling of CD44^+^/CD24^−/low^ and CD44^+/−^/CD24^+^ cell populations from breast cancer tissue, found expression of TGF*β* targets, such as VIMENTINE, CTGF, SERPINE1, SPARC, and TGFBR2, implying TGF*β* pathways seemed to be activated in these cells. Another recent study also has shown that gene expression signature of human mammary epithelial cell line introduced EMT inducers including TGF*β* strongly correlated with the signature derived from basal B cell lines of which subtype is characterized by a stem cell-like expression profile [54].

To induce complete EMT, TGF*β* works together with the Wnt, Hedgehog, Notch, and Ras signaling pathways, which are involved in the induction and maintenance of stem cell niches [55]. The canonical Wnt pathway, known to be a critical regulator of self-renewal in stem cell niches, is also implicated in the induction of EMT in cancer and is constitutively activated in colon, skin, and hematopoietic cancers [56–58]. 

In contrast, Tang et al. reported that TGF*β* inhibition increased the size of the CSC population and promoted tumorigenesis by a mechanism that was independent of direct effects on proliferation. In that study, which used transformed human breast epithelial cells, TGF*β* stimulation resulted in the loss of stem cell-like properties such as ability to form mammospheres [59]. Thus, further studies are needed to clarify these contradictory results on the role of TGF*β* signaling in the regulation of tumor-initiating property and EMT.

### 4.2. Circulating Tumor Cells

Identification of detectable circulating tumor cells (CTCs) in the peripheral blood of patients with solid tumors has been intensively studied for a decade, since CTCs may provide proof of principle for early primary cancer cell metastasis through the vascular network [60–62]. Several lines of evidence suggest that the finding of CTCs in the course of therapy possesses a consistent prognostic significance and is regarded as a predictive tool for response to treatments in cancer therapy [63, 64]. 

In this context, a recent study conducted in a cohort of 226 blood samples from 39 patients revealed that the majority of CTCs from metastatic breast cancer patients had EMT and CSC characteristics [65]. In that study, CTCs were found in 69 of 226 (31%) blood samples taken from patients with metastatic breast cancer to investigate the expression of EMT markers (Twist, AKT2, and PI3K*α*) and a stem cell marker (ALDH1). In the CTC-positive group, 62% were positive for the EMT markers and 69% for ALDH1, while in the CTC-negative group the percentages were 7% and 14%, respectively [65]. CTCs also showed reduced expression of epithelial-specific cytokeratins [66]. In disseminated tumor cells (DTCs), Twist, which is known as an EMT inducer, was overexpressed and the presence of Twist-positive cells in the bone marrow prior to chemotherapy has been significantly associated with relapse [67]. These results indicated that CTCs (or DTCs) might often exhibit characteristics of both EMT and CSCs, emphasizing their role in the formation of metastases. Therefore, further studies focused on identifying the features of cancer cells that have escaped from the primary tumor may allow the discovery of the critical mechanisms underlying cancer metastasis and recurrence.

### 4.3. Chemoresistance

It has been widely observed that many CSC populations are resistant to chemotherapy. One possible mechanism underlying chemoresistance is thought to arise from metabolic quiescence, a high level of expression of anti-apoptotic proteins [68, 69]. Another is the high expression of ATP-binding cassette (ABC) family genes, such as MDR1 and BCRP1, which could catalyze the efflux of a range of structurally unrelated anticancer drugs [70–72]. This property is shared by a subpopulation of normal stem cells known as “side population” (SP) cells. SP cells have been detected in several solid tumors, including breast, lung, and gastrointestinal cancers and have been shown to have the capacity for tumor initiation and self-renewal [71, 73–75]. Therefore, the identification of SP cells by flow cytometry is a promising method for the extraction of CSC populations in solid tumors. 

Induction of EMT also contributes to the decreased efficacy of chemotherapy in breast [76], colorectal [77], and ovarian cancer [78]. Introduction of Twist into breast cancer cells induces paclitaxel resistance and EMT, as well as AKT2 expression, which was amplified in breast cancer with acquired paclitaxel resistance [76]. A detailed characterization of cell systems reveals that mesenchymal derivatives of nonsmall cell lung cancer cells display an attribute of resistance to EGFR kinase inhibitor [79]. Creighton and colleagues reported in 2009 that a gene expression signature common to both CD44^+^/CD24^−/low^ and mammosphere-forming cells was found mainly in human breast cancer of the recently identified claudin-low molecular subtype, which is characterized by expression of many EMT-associated genes. They also demonstrated that residual breast cancer cell populations after conventional chemotherapy may be enriched for subpopulations of cells with both CSCs and mesenchymal features [13]. Consistently, CSCs, isolated using CD44^+^/CD24^−/low^ from human breast cancer, demonstrated resistance against the chemotherapeutic agents and the proportion of CD44^+^/CD24^−/low^ cells increased in breast cancer patients after treatment with anticancer drugs, including docetaxel, doxorubicin, and cyclophosphamide [80]. Of the 1% of cells that survived chemotherapy using paclitaxel or 5-fluorouracil, 30–35% of them were CD44^+^/CD24^−/low^, indicating selection of this population. 

On the basis of the above relationship between EMT and CSCs, using two populations of human mammary epithelial (HMLE) cells—one that had been induced EMT by knocking down of E-cadherin and one which had not—Gupta and colleagues screened a collection of about 16,000 compounds to find one showing selective toxicity towards the cells that had undergone EMT [81]. As the result of the screening, salinomycin was selected for further studies and was also discovered to suppress the proportion of CSCs that occur naturally as a subpopulation of breast cancer cells. Pre-treatment with paclitaxel was found to increase the tumor initiation ability more than 100 folds compared with pre-treatment of salinomycin.

These results point to the potential mechanisms of chemoresistance that allow CSC population increased by EMT to persistently survive and that may be responsible for recurrence in the primary or distant site following cancer treatment by chemotherapy after the majority of the cancer cells are killed. Taken together with the report by Mani et al. [12], these studies have led to the possibility that resistance to chemotherapy may be associated with the common characteristics of EMT and tumor-initiation abilities.

## 5. Conclusion

According to recent findings in this review, EMT may be a critical process underlying the subpopulation of cancer cells, CSCs, that are responsible for tumor initiation at metastatic sites and for regenerating the tumor after initial tumor regression by chemotherapy. This hypothesis may provide insight into current questions about the specific role of EMT in tumor metastasis. Therefore, identification of CSCs as specifically significant characteristics of tumor malignancy facilitated by EMT can help to provide an answer for this critical issue. Additional studies will be necessary in order to better establish and increase our understanding.

##  Conflict of Interests

The authors have no relevant conflict of interests.
